# Metagenomic detection and characterisation of multiple viruses in apparently healthy Australian *Neophema* birds

**DOI:** 10.1038/s41598-021-00440-1

**Published:** 2021-10-22

**Authors:** Subir Sarker

**Affiliations:** Molecular and Structural Virology Laboratory, Department of Physiology, Anatomy and Microbiology, School of Life Sciences, La Trobe University, Melbourne, VIC 3086 Australia

**Keywords:** Microbiology, Virology, Viral evolution

## Abstract

Emerging viral pathogens are a significant concern, with potential consequences for human, animal and environmental health. Over the past several decades, many novel viruses have been found in animals, including birds, and often pose a significant threat to vulnerable species. However, despite enormous interest in virus research, little is known about virus communities (viromes) in Australian *Neophema* birds. Therefore, this study was designed to characterise the viromes of *Neophema* birds and track the evolutionary relationships of recently emerging psittacine siadenovirus F (PsSiAdV-F) circulating in the critically endangered, orange-bellied parrot (OBP, *Neophema chrysogaster*), using a viral metagenomic approach. This study identified 16 viruses belonging to the families *Adenoviridae*, *Circoviridae*, *Endornaviridae*, *Picobirnaviridae* and *Picornaviridae*. In addition, this study demonstrated a potential evolutionary relationship of a PsSiAdV-F sequenced previously from the critically endangered OBP. Strikingly, five adenoviral contigs identified in this study show the highest identities with human adenovirus 2 and human mastadenovirus C. This highlights an important and unexpected aspects of the avian virome and warrants further studies dedicated to this subject. Finally, the findings of this study emphasise the importance of testing birds used for trade or in experimental settings for potential pathogens to prevent the spread of infections.

## Introduction

Over the past several decades, a significant effort has been made to better understand viromes in human and animals, but our understanding of the diversity of viruses in birds is not well understood. The vast majority of research aimed at describing viruses in birds has been on zoonotic viruses (e.g., avian influenza A virus)^1^, viruses that cause an economic loss in commercial poultry (e.g., Newcastle disease virus, infectious bronchitis virus)^2,3^, or viruses that result in notable mortality in wild birds (e.g., Wellfleet bay virus, and beak and feather disease virus)^4–8^. However, little is known about the viromes of birds that are neither economically important nor harbour any known zoonotic pathogens. There is a very limited understanding on the viromes of Australian birds chosen for trade or in vivo experiments, and order *Psittaciformes* (parrots) is the most traded group among avian orders^9^. The elegant parrot (*Neophema elegans*) and the scarlet-chested parrot (*Neophema splendida*) used in this study were purchased from a commercial trader without knowing any history of diseases.

Metagenomics, or metatranscriptomics, is a relatively new technique that enables the detection and characterisation of entire viromes in animals^10–12^, rather than on single species of virus in isolation. Before metagenomics, there was limited understanding of the viromes present in animal and human hosts. With the advent of metagenomics, information on eukaryotic and prokaryotic viruses, and even on viruses that infect other viruses, has increased^8,13–17^. However, little is known about the viruses that infect birds of the genus *Neophema*, an Australian genus with six parrot species, including one of the most critically endangered: the orange-bellied parrot (*Neophema chrysogaster*). Almost nothing is known about the viruses of the elegant parrot (*Neophema elegans*) and the scarlet-chested parrot (*Neophema splendida*) of the genus *Neophema*, a number of which were housed in La Trobe Animal Research and Teaching Facility for the ‘Parrot Genome Sequencing Project’. The handling of these birds may facilitate the transmission of infectious diseases and may serve as a source of exposure to other birds and to humans involved in their care^18,19^. Consequently, birds used for trade or in experiments may potentially cause the emergence of pathogens in captive birds, their dispersal into disease-free regions, and the transmission of zoonotic diseases.

In addition, a psittacine siadenovirus F (PsSiAdV-F) has recently been characterised from the critically endangered, orange-bellied parrot (*Neophema chrysogaster*)^20^. Subsequent phylogenetic analyses demonstrated that novel PsSiAdV-F infecting orange-bellied parrot evolved prior to all known members of siadenoviruses except the frog siadenovirus A, and did not show any obvious close evolutionary relationship^20^. Therefore, the aims of this study were not only to identify the known and unknown viruses that may exist in the two species of *Neophema* birds but also to track the evolutionary relationships of recently emerging PsSiAdV-F circulating in the critically endangered, orange-bellied parrot.

## Results

### Adenoviridae

Members of the family *Adenoviridae* are non-enveloped double-stranded DNA (dsDNA) viruses with a linear genome that ranges from about 26 kb to 46 kb^21–23^. They have been divided into six genera (*Aviadenovirus, Atadenovirus, Siadenovirus, Mastadenovirus, Ichtadenovirus* and *Testadenovirus*)^24–29^. They are recognised to cause a wide spectrum of illnesses, ranging from asymptomatic infections to severe illnesses and death in many animals and humans, and are integrated into surveillance programs given their importance in public health^30,31^. Multiple adenovirus sequences of the genus *Siadenovirus* and *Mastadenovirus* were detected in this study.

The complete genome of a psittacine siadenovirus F, PsSiAdV-F, detected in this study was a linear dsDNA molecule of 25,616 bp in length (coverage 127.58×), with a balanced G + C content (55.6%). Based on the complete genome identities, the known AdV genomes that were most closely related to the PsSiAdV-F strain S10/AU were PsSiAdV-F strain OBP2209 (PsSiAdV-F; 99.8%), followed by PsSiAdV-F strain WVL19065-01E (PsSiAdV-F; 99.7%), skua siadenovirus A (SuAdV-A; 57.0%) and raptor siadenovirus A (RAdV-A; 55.6%)^28^ (Supplementary Table S1). The PsSiAdV-F strain S10/AU genome had 30 predicted methionine-initiated open reading frames (ORFs) encoding proteins that were annotated as putative genes and were numbered from left to right (Table 1). Comparative analysis of the protein sequences encoded by the predicted ORFs, using BLASTX and BLASTP, identified homologs with significant protein sequence similarity for 25 ORFs (Table 1), while five ORFs (ORFs A-E) were found to be unique according to the BLAST database. Among the predicted protein coding ORFs of the PsSiAdV-F strain S10/AU genome, 25 were homologs to a recently sequenced PsSiAdV-F strain OBP2209 gene products (Table 1). The amino acid sequence similarity of predicted ORFs was very high compared to other siadenoviruses, ranging from 98.7 to 100% (Table 1).

**Table 1:** Predicted protein-coding genes of PsSiAdV-F strain S10/AU.

PsSiAdV-F strain S10/AUS	Start (nt)	Stop (nt)	Strand	Size (aa)	PsSiAdV-F strain OBP2209 Synteny	aa identity (%)
sialidase	150	1991	+	613	ORF01 sialidase	98.7
hypothetical protein	2003	2332	+	109	ORF02 hypothetical protein	100
IVa2	2361	3449	−	362	ORF03 IVa2	100
DNA polymerase	3439	6762	−	1107	ORF04 DNA polymerase	99.9
pTP	6759	8492	−	577	ORF05 pTP	100
52 K	8571	9443	+	290	ORF06 52 K	100
pIIIa	9433	10,929	+	498	ORF07 pIIIa	99.8
penton	10,951	12,291	+	446	ORF08 penton	100
pVII	12,292	12,681	+	129	ORF09 pVII	100
pX	12,683	12,859	+	58	ORF10 pX	100
pVI	12,877	13,527	+	216	ORF11 pVI	100
hexon	13,537	16,305	+	922	ORF12 hexon	100
protease	16,302	16,922	+	206	ORF13 protease	100
early E2	16,952	18,004	−	350	ORF14 early E2	100
ORFA	18,051	18,170	−	39		
100 K	18,209	20,203	+	664	ORF15 100 K	99.8
22 K	20,094	20,387	+	97	ORF16 22 K	100
33 K	20,589	20,687	+	32	ORF17 33 K	100
pVIII	20,748	21,209	+	153	ORF18 pVIII	100
E3	21,157	21,726	+	189	ORF19 E3	100
U exon	21,775	21,993	−	72	ORF20 U exon	100
fiber	21,992	23,023	+	343	ORF21 fiber protein	99.7
ORFB	22,974	23,102	−	42		
hypothetical protein	23,140	23,817	+	225	ORF22 hypothetical protein	99.6
hypothetical protein	23,828	24,352	−	174	ORF23 hypothetical protein	100
hypothetical protein	24,352	24,600	−	82	ORF24 hypothetical protein	100
ORFC	24,593	24,871	−	92		
hypothetical protein	24,868	25,395	−	175	ORF25 hypothetical protein	100
ORFD	25,386	25,493	−	35		
ORFE	25,448	25,615	−	55		

PsSiAdV-F, psittacine siadenovirus F; GenBank accession number of PsSiAdV-F strain OBP2209, MW365934; GenBank accession number of PsSiAdV-F strain S10/AU, MZ364296; aa, amino acid; nt, nucleotide.

**Figure 1:**
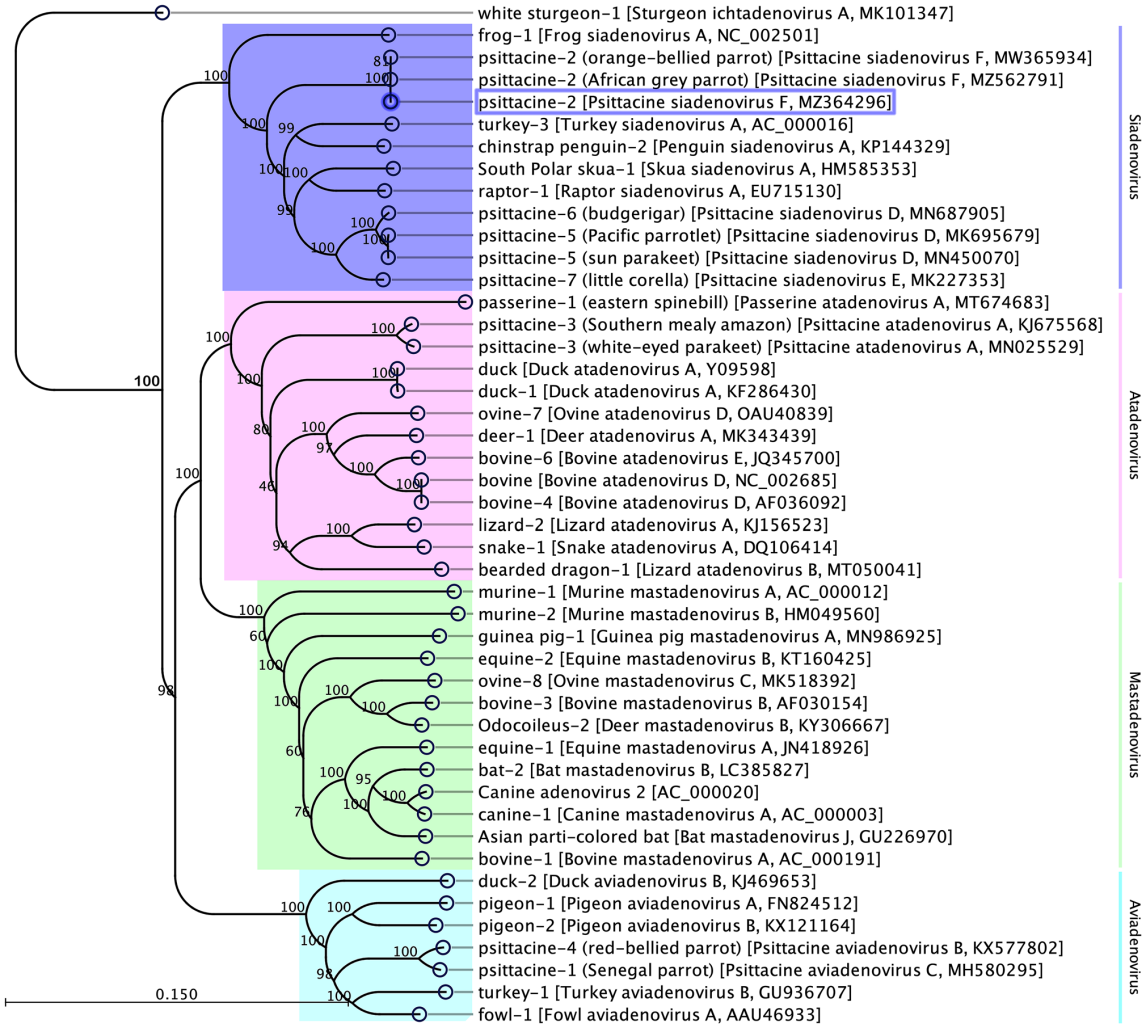
The phylogenetic tree shows the possible evolutionary relationship of PsSiAdV-F strain S10/AU with other selected AdVs. The maximum likelihood (ML) tree was constructed by using concatenated amino acid sequences of the complete DNA-dependent DNA polymerase, pTP, penton and hexon genes. Concatenated protein sequences were aligned with MAFTT (version 7.450)^65^ in Geneious (version 10.2.2, Biomatters, Ltd., Auckland, New Zealand), under the BLOSUM62 scoring matrix and gap open penalty = 1.53. The gap > 20 residues deleted from the alignments. The ML tree was constructed under the WAG substitution model, and 1000 bootstrap re-samplings using tools available in CLC Genomics Workbench (version 9.5.4). The numbers on the left show bootstrap values as percentages, and the labels at branch tips refer to original AdVs host species followed by AdVs name and GenBank accession numbers in parentheses. The clade correspondence to the genus *Siadenovirus* is highlighted in pink, and the PsSiAdV-F sequenced in this study is shown in the blue box.

Phylogenetic analyses based on two non-structural (polymerase and pTP) and two structural (penton and hexon) protein sequences clearly supported the inclusion of the newly sequenced PsSiAdV-F strain S10/AU in the genus *Siadenovirus*. In the resulting ML tree, based on concatenated amino acid sequences of four selected AdVs genes, the sequenced PsSiAdV-F strain S10/AU was positioned in a distinct subclade with other two PsSiAdV-F strains (e.g., OBP2209 and WVL19065-01E) (100% bootstrap support) (Fig. 1). Considering the genome-wide identities (> 99%) and phylogenetic position among three strains of PsSiAdV-F, it is reasonable to postulate that the PsSiAdV-F originates from the same ancestor.

Strikingly, this study detected five contigs that were shown to be homologs with human adenoviruses. The largest contig of 958 nt (coverage 8.25×, GenBank accession no. MZ364301) spanned part of the human adenovirus 2 penton gene. From further analysis, the human adenovirus 2 penton gene sequence had a > 99% (930/933) nucleotide match to EU128937.1 (Supplementary Table S2). At the amino acid level, this segment had a 296/298 (99%) amino acid match to the penton gene of the human adenovirus 2 (GenBank accession no. CAC67478.1). Phylogenetically the detected penton gene of the human adenovirus 2 occupied the same clade dominated by other human mastadenoviruses (Fig. S1). Another contig of 322 nt (coverage 4.60×, GenBank accession no. MZ364305) was also part of the adenovirus penton gene and showed the highest nucleotide identity (98.8%) with another human mastadenovirus C (GenBank accession no. AHN92668.1). There were three further contigs of 435, 430 and 405 nt (coverage 6.41×, 8.19× and 5.27×, respectively; GenBank accession no MZ364303, MZ364302 and MZ364304, respectively) spanning part of the human mastadenovirus C capsid protein precursor pIIIa gene, encapsidation protein 52 K gene and capsid protein precursor pVI gene, respectively. The similarities of assembled sequences were found to be a 430/435 (98.9%) nucleotide match to the human mastadenovirus C (GenBank accession no. MH121111.1) (Table S3 and Fig. S2), a 386/390 (99.0%) nucleotide match to the human mastadenovirus C (GenBank accession no. MN088492.1) (Table S4), and a 403/403 (100%) nucleotide match to the human mastadenovirus C (GenBank accession no. MN088492.1), respectively.

### Circular viruses

#### Circoviridae

**Figure 2:**
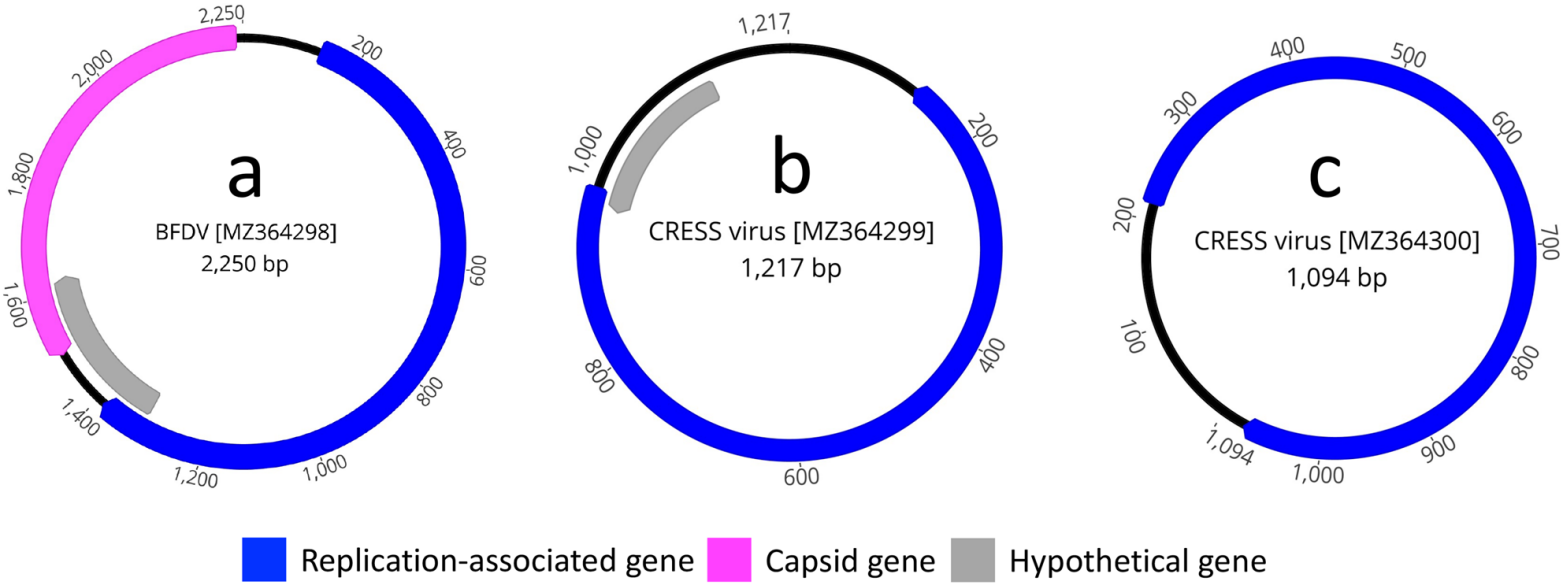
The whole genome sequence structure of circular viruses isolated from *Neophema* bird species. The arrows symbolise genes and open reading frames (ORFs), with orientation indicating their direction of transcription. Each gene or ORF is colour-coded, as indicated by the key in the legend.

Beak and feather disease virus (BFDV) is a member of the *Circoviridae* family and has a relatively simple but compact circular, ambisense single-stranded DNA (ssDNA) genome of approximately 2.0 kb encoding a replicase (Rep) and a single capsid protein (Cap) which facilitates whole-genome viral epidemiological analysis^6,7,32,33^. BFDV is highly genetically diverse and infects a large number of psittacine and non-psittacine bird species^5,33–39^. A complete genome sequence of BFDV (2250 nt in length, coverage 171.10×) was detected in this study (Fig. 2a). As shown in Fig. 2a, the genome of BFDV (GenBank accession no. MZ364298) contained two bidirectional ORFs encoding the putative Rep and Cap proteins. A BLASTP search in GenBank based on the protein sequence of Rep showed the highest identity of 98.27% (query coverage, 69%; E-value, 0.0) to a BFDV isolated from an Australian psittacine bird, the long-billed corella (*Cacatua tenuirostris*), in 2010 (GenBank accession no. KF385420.1)^7^, whereas Cap showed the highest identity of 96.76% (query coverage, 100%; E-value, 7.0 × 10^−177^) to a BFDV isolated from an Australian psittacine bird, the little corella (*Cacatua sanguinea*), in 2014 (GenBank accession no. KY189059.1). Phylogenetic analysis based on selected completed genome sequences of BFDV showed that the sequenced BFDV strain in this study clustered in a distinct subclade with other BFDVs isolated from Australian cockatoos (Fig. S3), which suggests that the BFDV sequenced in this study is likely to have originated from the same ancestor.

### CRESS-DNA viruses

Two complete CRESS-DNA genomes of 1217 nt (coverage 27.40×, Fig. 2b) and 1094 nt (coverage 4.80×, Fig. 2c) in length were detected in this study. As shown in Fig. 2 (b and c), both the CRESS-DNA genomes encoded a putative Rep gene, while CRESS-DNA isolate001 also contained an additional hypothetical gene. A BLASTP search in GenBank, based on the protein sequence of Rep of CRESS-DNA isolate001, showed the highest identity of 80.94% (query coverage, 99%; E-value, 3.0 × 10^−171^) to a CRESS-DNA virus isolated from seabass tissue in the United States of America in 2017 (accession no. AXQ65592.1), whereas Rep of CRESS-DNA isolate002 showed the highest identity of 80.92% (query coverage, 100%; E-value, 9.0 × 10^−175^) to a CRESS-DNA virus isolated from seabass tissue in the United States of America in 2017 (accession no. AXQ65592.1). At the genomic level, both the CRESS-DNA genomes sequenced in this study showed highest nucleotide identity (84.27%) with a CRESS-DNA virus isolated from seabass tissue in the United States of America in 2017 (accession no. MH648902.1). Phylogenetically both the CRESS-DNA virus detected in this study positioned in a subclade with other CRESS-DNA viruses sequenced from seabass and dragonfly (*Procordulia grayi*)^40^ in the United States of America and New Zealand, respectively (Fig. S4).

### Endornaviridae

The family *Endornaviridae* includes viruses with linear, single-stranded, positive-sense RNA genomes that range from 9.7 to 17.6 kb and have been reported as infecting plants, fungi and oomycetes^41^. In this study, two fragments of psittacine alphaendornavirus (contigs of 697 nt, and 613 nt in length, coverage 4.83× and 4.93×, GenBank accession no. MZ561459 and MZ561460, respectively) were detected. Both the fragments of psittacine alphaendornavirus showed the highest nucleotide identities (> 99.0%, Figs. S5 and S6) with the polyprotein of *Helianthus annuus alphaendornavirus* (GenBank accession no. NC_040799.1), which was isolated from sunflowers (*Helianthus annuus*) in China in 2016. Phylogenetically, two fragments of a psittacine alphaendornavirus detected in the *Neophema* birds were evolutionarily linked with the alphaendornaviruses isolated from sunflowers (*Helianthus annuus*) in China (Fig. S7).

### Picobirnaviridae

Viruses of the family *Picobirnaviridae* are non-enveloped, bi-segmented, double-stranded RNA (dsRNA) viruses of approximately 2.4 to 2.7 kb in length, which contain two functional genes, a structural capsid gene and an RNA-dependent RNA polymerase (RdRp) gene^42^. Evidence of picobirnavirus (PBV) sequences have been reported in humans, invertebrates, environmental water samples and a wide range of animals, including birds worldwide^12,42–47^. In this study, two contigs of 444 nt and 345 nt in length (coverage 4.86× and 11.15×, respectively), were detected and were found to be psittacine picobirnaviruses. The contig of 444 nt spans part of the RdRp gene of the human picobirnavirus sp. (GenBank accession no. KU892528.1); however, it showed only a 7% query sequence coverage with identities of 100%. Another contig of 345 nt had a 100% nucleotide match (query coverage of 8%) to the capsid protein gene of the human picobirnavirus sp. (GenBank accession no. KU892525.1). However, this study was unable to detect any protein sequence similarity of the detected contigs using a BLAST search. In the resultant phylogenetic tree, the psittacine picobirnaviruses positioned in a subclade dominated by picobirnaviruses sequenced from Australian Shelduck^45^, chickens^48^ and humans (Fig. S8). However, bootstrap support for most of nodes in this phylogeny was poor and further sequencing followed by clarification of the evolutionary relationship among these viruses is required.

### Picornaviridae

**Figure 3:**
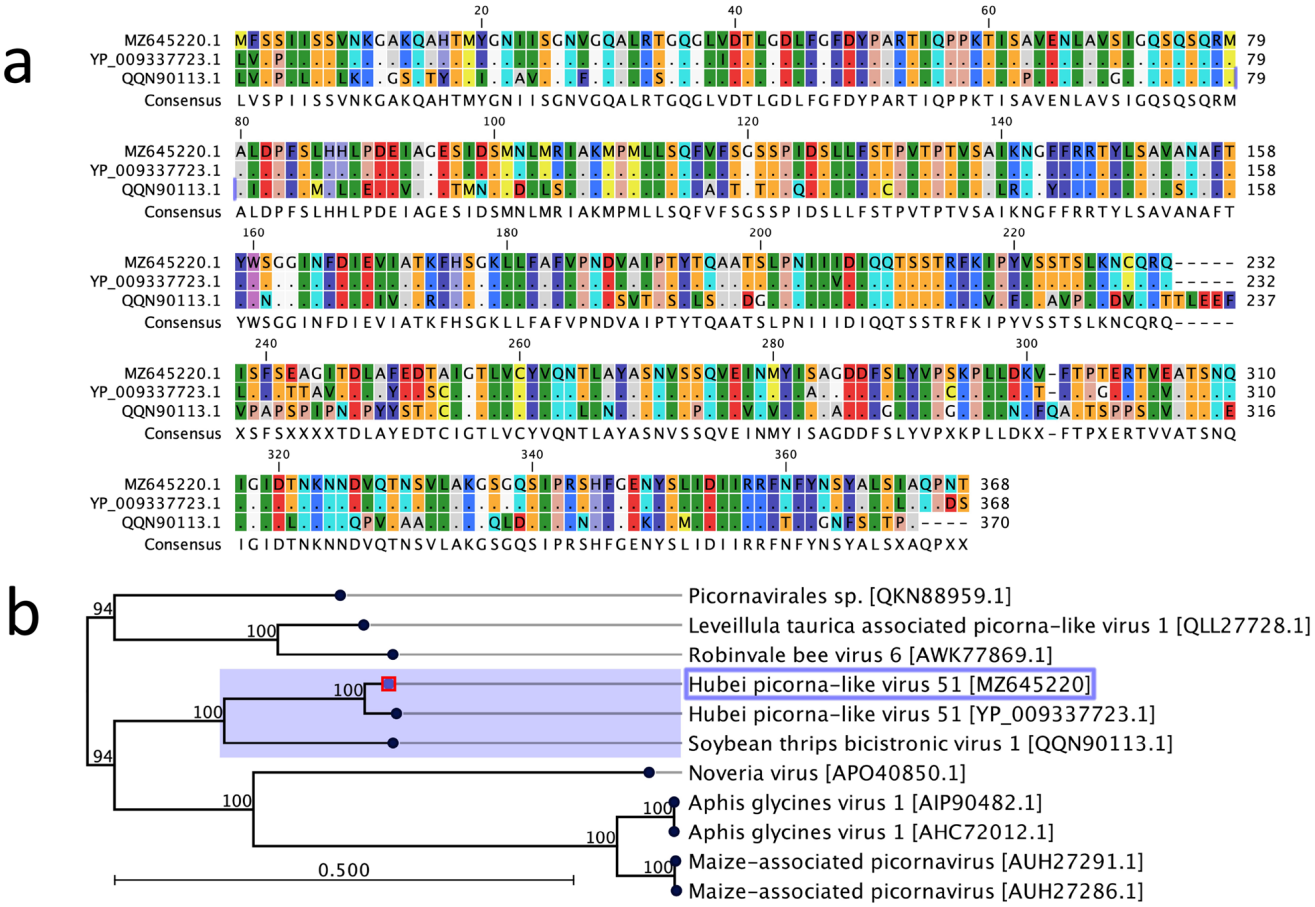
Comparison of picorna-like virus detected in this study (GenBank accession no. MZ645220) with other selected picorna-like viruses of the family *Picornaviridae*. (**a**) Multiple alignments of the selected amino acid sequences were performed using tools available in CLC Genomics Workbench (version 9.5.4), where dots represent those amino acids identical to the picorna-like virus detected in this study. (**b**) A maximum likelihood (ML) tree was constructed by using selected amino acid sequences of the partial hypothetical protein 1 gene of Hubei picorna-like virus. The ML tree was constructed under the WAG substitution model, and 1000 bootstrap replicates using tools available in CLC Genomics Workbench (version 9.5.4). The numbers on the left show bootstrap values as percentages, and the labels at branch tips refer to original picorna virus names, followed by GenBank accession numbers in parentheses. The clade correspondence to the picorna-like virus sequence from this study is highlighted in purple, and the picorna-like virus sequenced in this study is shown in the purple box.

Picornaviruses are non-enveloped, positive-sense, single-stranded RNA viruses, which infect many vertebrates, including mammals and birds. Recent studies have detected several picorna-like viruses from Australian wild birds^12^. In this study, a contig of 1135 nt in length (coverage 10.17×, GenBank accession no. MZ645220) was detected, which was part of the hypothetical protein 1 gene of Hubei picorna-like virus (GenBank accession no. KX883953.1) and showed the highest nucleotide identity (94.03%) between them. This contig had a 94.6% match to the hypothetical protein 1 gene of Hubei picorna-like virus at the amino acid level, followed by a 69.7% match to the structural polyprotein of soybean thrips bicistronic virus 1 (GenBank accession no. QQN90113.1) (Fig. 3a). A phylogenetic tree, using the hypothetical protein 1 gene of Hubei picorna-like virus, showed that the picorna-like virus detected in this study occupied the same clade as Hubei picorna-like virus 51 (Fig. 3b), sequenced from flying insects^13^.

## Discussion

This study used a viral metagenomic approach to help determine viral diversity in apparently healthy captive psittacine birds housed for the ‘Parrot Genome Sequencing Project’. Many captive birds are sourced from the wild and are used for various in vivo experiments, but their virome structure is largely unknown. Accordingly, this study identified many known and unknown viruses in the families *Adenoviridae*, *Circoviridae*, *Endornaviridae, Picobirnaviridae* and *Picornaviridae*, as well as unclassified CRESS-DNA viruses, in apparently healthy Australian birds of the genus *Neophema*.

The main aims of this study were not only to understand the virome structure of apparently healthy Australian *Neophema* birds but also to track the evolutionary history of a recently sequenced PsSiAdV-F in the critically endangered Australian parrot, the orange-bellied parrot (*Neophema chrysogaster*)^20^. Notably, the PsSiAdV-F sequenced from this study was very similar (a nucleotide identity of > 99% at genome level) to the recently sequenced two PsSiAdV-F strains of OBP2209 and WVL19065-01E from the orange-bellied parrot and African grey parrot (*Psittacus erithacus*), respectively, and phylogenetically all the three strains of PsSiAdV-F were positioned in a monophyletic subclade (100% bootstrap support**)** (Fig. 1) that is basal to all known avian siadenoviruses. This suggests that all the avian siadenoviruses evolved from the ancestral bird that gave rise to PsSiAdV-F, which is consistent with a recent study that reported that PsSiAdV-F was basal to all avian siadenoviruses^20^. Evidence that this ancestral species may have been a primitive Australian or African parrot includes the observation that PsSiAdV-F has only been detected in psittacine species where it causes predominantly subclinical infections. The PsSiAdV-F is also widespread in captive populations of at least three species of Australian parrot: the orange-bellied parrot (*Neophema chrysogaster*), the scarlet-chested parrot (*Neophema splendida*) and the Bourke’s parrot (*Neopsephotus bourkii*)^20,49,50^. The finding that PsSiAdV-F originated in Australian psittacine birds needs to be confirmed by it being identified in other Australian psittacine species, and, to date, surveys of psittacine birds in Australia have not detected it.

Except for a recent study in which the authors reported a mastadenovirus sequence (a variant of murine adenovirus 2) in Australian wild birds species^26^, evidence of mammalian adenoviruses (such as human adenovirus 2 and human mastadenoviruses) in avian host species is scarce. For the first time, this study reported five different contigs, which were found to be highly similar (> 99% identity) to the human adenovirus 2 and human mastadenoviruses. It is impossible to elucidate the host–pathogen dynamics of mammalian adenoviruses from this case alone, but it is evident that captive birds have close interaction with humans. This, therefore, suggests that these birds are potentially exposed to viruses from humans and other animal species, which requires further investigation.

Circoviruses are small, non-enveloped icosahedral viruses with circular ssDNA genomes of only 2 kb, making them the smallest autonomously replicating pathogens. Numerous studies have shown that all Psittaciformes, including threatened Australian psittacine bird species, can be infected by a circovirus (e.g., beak and feather disease virus, BFDV)^5,6,33,37,51,52^. Therefore, the finding of the BFDV genome in this study supports the presence of BFDV as an endemic virus in Australian birds. In addition, the CRESS-DNA virus has a small circular rep-encoding ssDNA (CRESS-DNA) genome that encodes a replication-associated protein (Rep)^53,54^. However, there is lack of adequate information on the existence of the CRESS-DNA virus, as well as the pathology they may develop in psittacine birds. This study found evidence of two divergent CRESS-DNA virus genomes, which were evolutionarily related with the CRESS-DNA virus isolated from seabass tissue in the United States of America in 2017 (nucleotide identity ranging from 80 to 84%), and this study cannot rule out that they may also represent food contaminants and environmental pollution. In the absence of any CRESS-DNA virus sequence from Australian psittacine birds, this study suggests that more structured sampling that includes additional hosts could help better understand the ecology and epizootiology of potentially pathogenic CRESS-DNA viruses.

Birds may serve as reservoirs, intermediate hosts or transporters of parasites and pathogens^11,12,55^. The similarity of some of the detected sequences of the avian and non-avian host-associated RNA viruses to the viruses that were characterised in China (e.g., *Endornaviridae*)^13^, and Belgium (e.g., *Picobirnaviridae*), highlights that birds may harbour a diverse range of viruses that need to be investigated further. It is also noteworthy to mention that during the BLASTN and BLASTX search, none of the top-matched sequences were similar to or related to those viruses from Australia.

Using viral metagenomic or meta-transcriptomic approaches, picornaviruses were previously reported in wild birds without understanding any pathology they may cause^11,12,56,57^. However, information on picornaviruses harboured by apparently healthy captive birds is scarce. The picornavirus detected in this study was shown to be closely phylogenetically related to a Hubei picorna-like virus 51 sequenced from flying insects^13^ (Fig. 3b). While further work is required to understand the ecology and pathogenicity of the viruses detected in this study, numerous studies have suggested that the presence of immunosuppressive diseases such as BFDV may increase the likelihood that subclinical infections by other viruses progress to clinical disease in birds infected with multiple agents^58,59^. In addition, BFDV infection is often associated with clinical evidence of acquired immunodeficiency, leading to a variety of secondary or opportunistic infections^18,60,61^.

The findings of non-avian host-associated viruses detected in this study were endornavirus and picornavirus, which agree with the previous reports by others, where a large number of non-avian associated viruses were detected in Australian birds^11,12^. Some aspects of this case are difficult to explain fully without conducting ethically debatable experimental virus-transmission experiments. A possible scenario for transmission could be ingestion of insect vector contaminated food. Alternatively, they may have been eaten infected insects before entering into the pet- trade. Moreover, this may also indicate that many diverse viruses may be harboured by the closely related avian host species, not detected yet.

## Conclusions

Overall, this study demonstrates a significant viral diversity in *Neophema* birds. Notably, it shows the evidence of genetically and phylogenetically similar psittacine siadenovirus (PsSiAdV-F) circulating between critically endangered orange-bellied parrots and other *Neophema* bird species. This study demonstrates that apparently healthy captive birds can sustain a significant viral diversity. Furthermore, the presence of viral pathogens in these birds presents a concerning example of the ease with which such infectious agents may spread to other captive birds, and the humans in their proximity. Finally, the findings of this study emphasise the importance of testing captive birds for possible pathogens to prevent the spread of potentially deadly diseases.

## Methods

### Sampling and ethical approval

In 2020, four fresh faecal samples were collected from two different species, the elegant parrot (*Neophema elegans*) and the scarlet-chested parrot (*Neophema splendida*) that were housed in La Trobe Animal Research and Teaching Facility for the ‘Parrot Genome Sequencing Project’. These samples were stored at − 80 °C within 1 h of their collection and kept in these conditions until their processing. Bird sampling was obtained following approved guidelines set by the Australian Code of Practice for the Care and Use of Animals for Scientific Purposes and approved by the La Trobe University Animal Ethics Committee (research permit number AEC19035) and Department of Environment, Land, Water and Planning (permit number 10009300).

### Virus enrichment and virus nucleic acid extraction

Elimination of potential impurities, such as host cells, bacteria, food particles and free nucleic acids, from the faecal samples, followed by enrichment of virus particles was performed as per stated methods^12^, with miror variations. Briefly, the faecal materials were aseptically resuspended and homogenised vigorously in sterile phosphate-buffered saline (PBS) (1:10) and centrifuged at 2500 × *g* for 90 min at 4 °C. The supernatant was filtered using a 0.80 μm syringe filter and the filtrate processed downstream. The samples were then ultracentrifuged at 178,000 g for 1 h (30 psi for 1 h) at 4 °C using the Hitachi Ultracentrifuge CP100NX. The supernatant was discarded, and the pellet was suspended in 130 µL of sterile PBS. The filtrates were then nuclease-treated using 2 µL of benzonase nuclease (25–29 U/µL, purity > 90%, Millipore) and 1 µL of micrococcal nuclease (2,000,000 gel units/mL, New England Biolabs) and incubated at 37 °C for 2 h. The nuclease reaction was stopped by adding 3 µL of 500 mM ethylenediaminetetraacetic acid (EDTA). Viral nucleic acids were extracted using the QIAamp Viral RNA Mini Kit (Qiagen, Valencia, CA, USA), without adding any carrier RNA, which allowed the extraction of both viral DNA and RNA simultaneously. The quantity and quality of the isolated nucleic acids were determined using Nanodrop and an Agilent Tape Station (Agilent Technologies, Mulgrave, VIC, Australia) by the Genomic Platform, La Trobe University.

### Next-generation sequencing

Before library construction, extracted nucleic acids were subjected to cDNA synthesis, and amplification was carried out using the Whole Transcriptome Amplification Kit (WTA2, Sigma-Aldrich, Darmstadt, Germany) as per manufacturer instructions. Amplified PCR products were then purified using the Wizard® SV Gel and PCR Clean-Up kit (Promega, Madison, WI, USA). The quantity and quality of the purified product were checked using a Qubit dsDNA high sensitivity assay kit with Qubit Fluorometer v3.0 (Thermo Fisher Scientific, Waltham, MA, USA).

The library construction was performed as a pool that contained four samples using the Illumina DNA Prep (Illumina, San Diego, CA, USA) as per kit instructions, starting with 250 ng of DNA as measured by Qubit (Invitrogen). The quality and quantity of the prepared library was assessed by the Australian Genome Research Facility (AGRF), Melbourne, Australia. The prepared library was normalised and pooled in equimolar quantities. The quality and quantity of the final pooled library were further assessed as described above before sequencing by the facility. According to the manufacturer’s instructions, cluster generation and sequencing of the pooled library was performed with read lengths of 150-bp paired-end on Illumina® NovaSeq chemistry.

### Bioinformatic analyses

The resulting 52.1 million raw sequence reads were used for a quality control check using CLC Genomics Workbench (version 9.5.4). Preliminary quality evaluation for all raw reads was generated, pre-processed to remove ambiguous base calls and poor-quality reads and trimmed to remove the Illumina adapter sequences. Trimmed sequence reads were mapped against the chicken genome (*Gallus*, GenBank accession number NC_006088) to remove likely host DNA contamination. In addition, reads were further mapped to *Escherichia coli* bacterial genomic sequence (GenBank accession no. U00096) to remove possible bacterial contamination. Unmapped reads were used as input data for de novo assembly using a SPAdes assembler (version 3.10.1)^62^ under the ‘careful’ parameter in the LIMS-HPC system (a High-Performance Computer specialised for genomics research in La Trobe University). The resulting contigs were compared against the nonredundant nucleotide and protein databases on GenBank using BLASTN and BLASTX^63^, respectively, with an E-value threshold of 1 × 10^−5^ to remove potential false positives. Contigs that were significant BLAST hits with bacteria, eukaryotes or fungi were filtered out to remove non-viral reads. Virus contigs of interest greater than 300 nucleotides (nt) were imported in Geneious software (Biomatters Ltd., New Zealand, version 10.2.2) for further functional analysis. Average coverage of the viral contigs were calculated using the clean raw reads in CLC Genomics Workbench (version 9.5.4).

### Functional annotations

The detected complete genome of adenovirus was annotated as per stated protocol^23^ using Geneious software (version 10.2.2, Biomatters, New Zealand). Briefly, open reading frames (ORFs) longer than 30 amino acids, with a methionine start codon (ATG) and minimal overlap with other ORFs (not exceeding 50% of one of the genes), were selected and annotated. All the detected circovirus genomes were annotated using Geneious software (version 10.2.2, Biomatters, New Zealand), where representative circovirus genomes were used as reference guidelines^6,7,38,64^. Similarity BLAST searches were performed on the predicted ORFs and were annotated as potential genes if predicted ORFs showed significant sequence similarity to known viral or cellular genes (E-value threshold of 1 × 10^−5^)^63^. Detected partial genomes or genes content were annotated according to the BLASTN and BLASTX^63^ search results.

### Comparative genomics and phylogenetic analyses

Genomic features of the newly sequenced viral genomes were visualised using Geneious (version 10.2.2). Sequence similarity percentages between representative viruses were determined using tools available in Geneious (version 10.2.2).

For phylogenetic analyses, representative viral genome or gene sequences were downloaded from GenBank, and virus-specific trees were constructed using CLC Genomics Workbench (version 9.5.4) and Geneious software (version 10.2.2, Biomatters, New Zealand). Amino acid sequences of protein-coding genes and nucleotide sequences of the selected partial genes were aligned using the MAFTT L-INS-I algorithm implemented in Geneious (version 7.388)^65^. To determine the best-fit model to construct phylogenetic analyses, a model test was performed using CLC Genomics Workbench (version 9.5.4) using default parameters, favouring a general-time-reversible model with gamma distribution rate variation and a proportion of invariable sites (GTR + G + I). Phylogenetic analyses for nucleotide and protein sequences were performed using the GTR and WAG substitution model, respectively, with 1000 bootstrap support in CLC Genomics Workbench (version 9.5.4).

## Supplementary Information

Supplementary Information.

## Data Availability

All sequences analysed have been deposited in NCBI GenBank under the accession numbers MZ364296, MZ364298-MZ364305, MZ561459-MZ561460, MZ645220. Raw sequencing data from this study has been deposited in the NCBI Sequence Read Achieve (SRA) under the BioProject ID: PRJNA750905 (BioSample accessions: SAMN20500864) (http://www.ncbi.nlm.nih.gov/sra/).
