# Supplementary material for: Metagenomic detection and characterisation of multiple viruses in apparently healthy Australian Neophema birds
Source: Sci Rep. 2021 Oct 22;11:20915. doi: 10.1038/s41598-021-00440-1 (PMC8536680; doi:10.1038/s41598-021-00440-1)
Supplement: Supplementary file 1 — Supplementary Information. [file 41598_2021_440_MOESM1_ESM.pdf]

Metagenomic detection and characterisation of multiple viruses in apparently healthy Australian *Neophema* birds

Subir Sarker [1*]

1 Molecular and Structural Virology Laboratory, Department of Physiology, Anatomy and Microbiology, School of Life Sciences, La Trobe University, Melbourne, VIC 3086, Australia.

[*]**Author for correspondence:** Dr Subir Sarker, Molecular and Structural Virology Laboratory, Department of Physiology, Anatomy and Microbiology, School of Life Sciences, La Trobe University, Melbourne, VIC 3086, Australia, email: s.sarker@latrobe.edu.au; phone: +61 3 9479 2317; fax: +61 3 9479 1222.

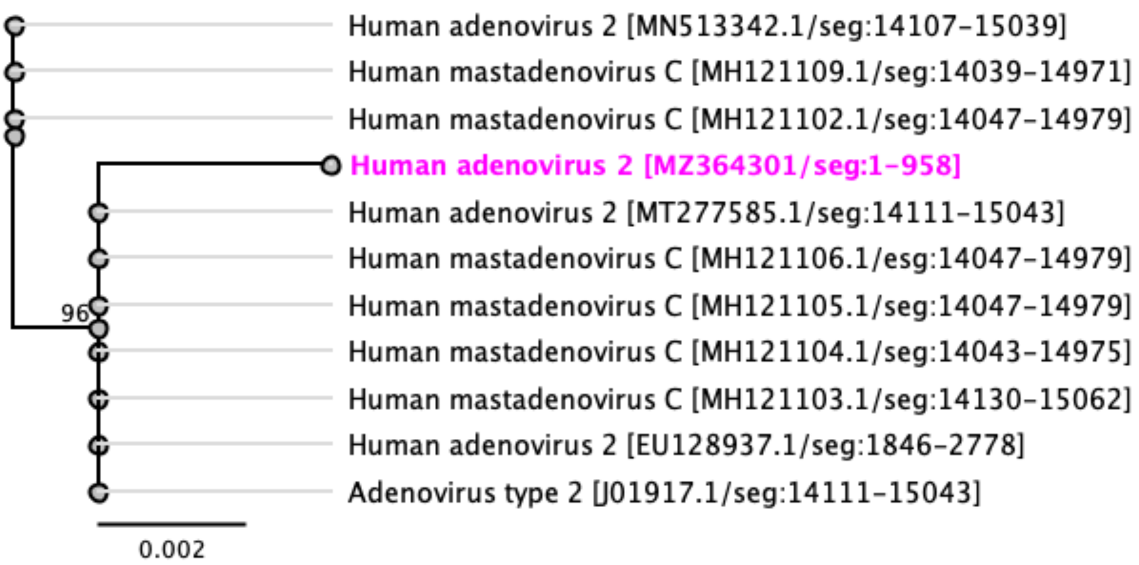

Figure S1. Phylogenetic tree showed the possible evolutionary relationship of partial penton gene of human adenovirus 2 detected in this study with other selected AdVs. Selected partial nucleotide sequences of penton gene were aligned with MAFTT (version 7.450) (Katoh and Standley, 2013) in Geneious (version 10.2.2). The unrooted Maximum likelihood (ML) tree was constructed with PhyML (Guindon et al., 2010) under the GTR substitution model with 1000 bootstrap resamplings using tools available in Geneious (version 10.2.2). The numbers on the left shown bootstrap values as percentages and the labels at branch tips refer to original AdVs species name followed by GenBank accession number and matched segment position of the gene in parentheses. The human adenovirus 2 detected in this study was shown in pink colour.

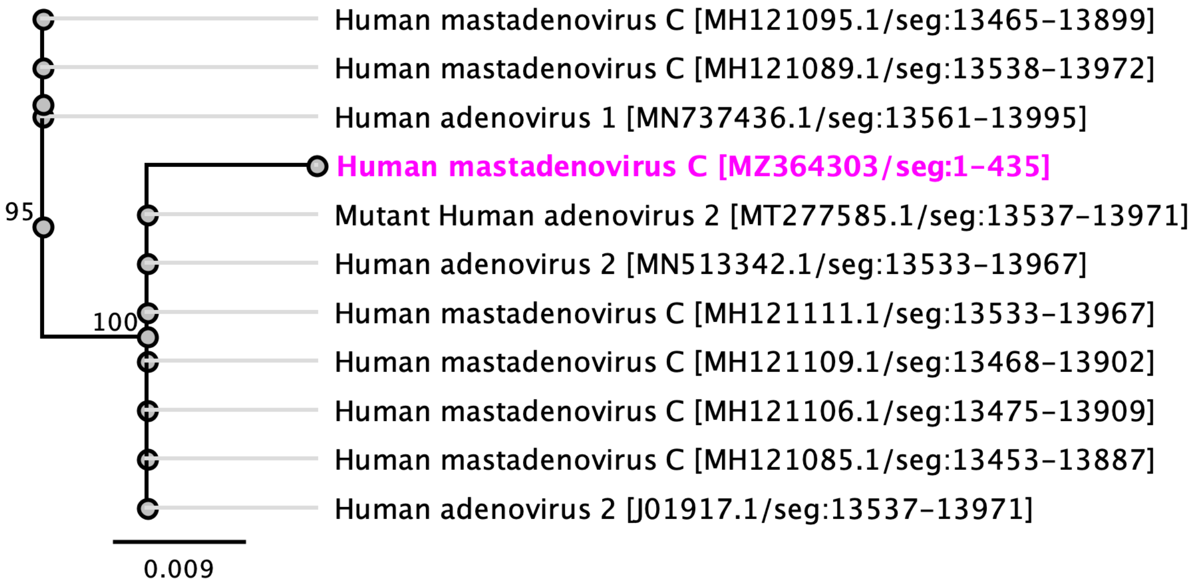

Figure S2. Phylogenetic tree showed the possible evolutionary relationship of partial capsid protein precursor pIIIa gene of human mastadenovirus C detected in this study with other selected AdVs. Selected partial nucleotide sequences of penton gene were aligned with MAFTT (version 7.450) (Katoh and Standley, 2013) in Geneious (version 10.2.2). The unrooted Maximum likelihood (ML) tree was constructed with PhyML (Guindon et al., 2010) under the GTR substitution model with 1000 bootstrap resamplings using tools available in Geneious (version 10.2.2). The numbers on the left shown bootstrap values as percentages and the labels at branch tips refer to original AdVs species name followed by GenBank accession number and matched segment position of the gene in parentheses. The human adenovirus 2 detected in this study was shown in pink colour.

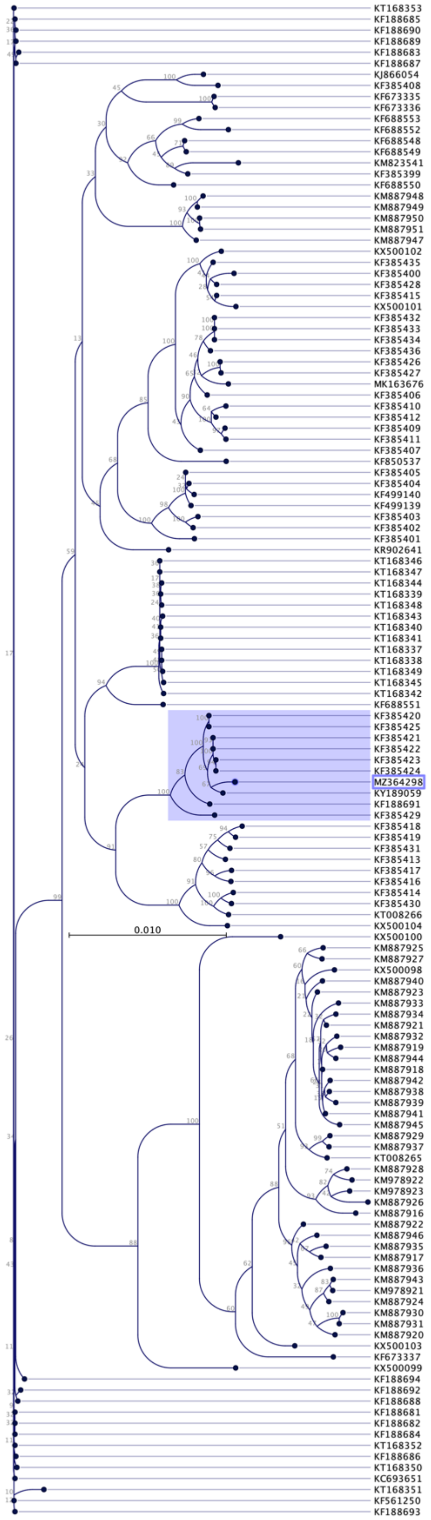

Figure S3. Phylogenetic tree showed the possible evolutionary relationship of complete genome of beak and feather disease virus (BFDV) detected in this study with other selected BFDV. Selected nucleotide sequences of complete BFDV genomes were aligned with MAFTT (version 7.450) (Katoh and Standley, 2013) in Geneious (version 10.2.2). The unrooted ML tree was constructed under the GTR substitution model, and 1000 bootstrap re-samplings were chosen to generate ML trees using tools available in CLC Genomics Workbench (version 9.5.4). The numbers on the left show bootstrap values as percentages, and the labels at branch tips refer to original GenBank accession number. The subclade related to this study is highlighted as blue background colours, and the BFDV sequenced in this study is shown in blue box.

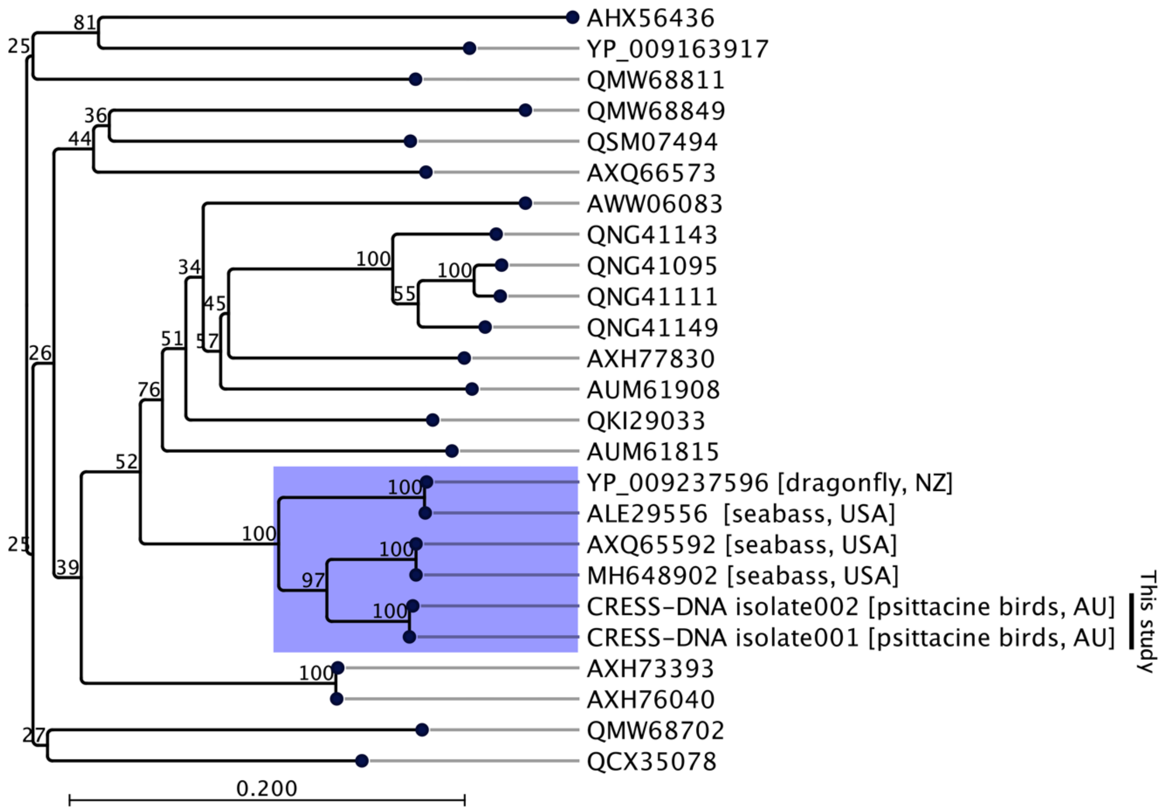

Figure S4. Phylogenetic tree showed the possible evolutionary relationship of CRESS-DNA virus detected in this study with other selected CRESS-DNA virus using replication associated protein coding gene. Amino acids sequences of complete replication associated protein gene were aligned with MAFTT (version 7.450) (Katoh and Standley, 2013) in Geneious (version 10.2.2). The ML tree was constructed under the WAG substitution model and 1000 bootstrap re-samplings using tools available in CLC Genomics Workbench (version 9.5.4). The numbers on the left show bootstrap values as percentages, and the labels at branch tips refer to original GenBank accession numbers followed by isolating host and country abbreviation in parentheses. The subclade related to this study is highlighted as blue background colours.

```
Score            Expect   Identities      Gaps       Strand
1266 bits(685)   0.0      693/697(99%)    0/697(0%)  Plus/Minus

Query  1     GAGCTGTCCAACCCCTCCCATAGCATATCCTGCAATCTATTCCTCTACCGTTTCTTTTGT  60
Sbjct  9969  .....T............T.........................................  9910

Query  61    TTCCGTAGTCTGTGTCAACTTTCCTAGTGTTCTTTGCGGCATAGTATAACCACTTTGACT  120
Sbjct  9909  ............................................................  9850

Query  121   CCGATATTATCTGTTCTTGACCTGCTAGTGTTTTAATCTCTGTTGCCTTGTATGGTTTCA  180
Sbjct  9849  ............................................................  9790

Query  181   TCTGTTCGTGAAGTATCTCTCCATGTGCATGGTACGCTAATGTGGCTGTACTGGGTGGCC  240
Sbjct  9789  ............................................................  9730

Query  241   CTCTATTGGTGTCGAATGGAAGCACGGGTTGGATTATAGTGTGTTCTTTTGCCACATATA  300
Sbjct  9729  ............................................................  9670

Query  301   CAAACCTTGGTTTCCTTACGTTTTCCAAATTGCAAACTATTAGCGTATCGCTATTCTCGG  360
Sbjct  9669  ............................................................  9610

Query  361   CCAATGTGACGTCTATTGATCCGTAAGATCCACAACATGTGTAGTGTATTCTTCTTGGCT  420
Sbjct  9609  ............................................................  9550

Query  421   CCGTGATTTCAAAGTTCCACTCCTTCATTAGGGGTTTCATTTCCTCTTGTAGCTTAAAGT  480
Sbjct  9549  ............................................................  9490

Query  481   ACTTAGATTTGTTAGGTACATCGTCTGTGAAGGTGGGCCCTTGCGTTGTTATGGCGGGTC  540
Sbjct  9489  ............................................................  9430

Query  541   TTTGGGGTAGTGATTTACTTGAGAAGATTTCTTGGTCAAGAGGCGGGCTCAAATTTGTTA  600
Sbjct  9429  ............................................................  9370

Query  601   TCTTTTCGTATGTTGAGGGGTTGGTTCTCAGCATGTCCATCTGTGTTGCCAAACTTCTTA  660
Sbjct  9369  ...................................A........................  9310

Query  661   GTCCTACTATTAGATTTTTCGCAACCCCTGTGACCAG  697
Sbjct  9309  ......T..............................  9273
```

Figure S5. Nucleotide identities between psittacine alphaendornavirus isolate001 sequenced in this study and Helianthus annuus alphaendornavirus (GenBank accession no. NC_040799.1).

```
Score            Expect   Identities     Gaps        Strand
1107 bits(599)   0.0      603/605(99%)   0/605(0%)   Plus/Minus

Query  1      TAACACGATGGGTCCATTATATTTCAGTGACTCTATGACTTCCGGTTCCAGAGGTTTCAT  60
Sbjct  11366  ............................................................  11307

Query  61     AGTTTGGTCTACCAGGAATTGCCTGGCTTTCTCAATGTTCACTGCTACTATCTCATTGTG  120
Sbjct  11306  ............................................................  11247

Query  121    GTGGTTTCTGTTCCGGAGGTCAGTTAGTATAACTTTCATTCCCTTTAGGCTTATTGGTAG  180
Sbjct  11246  ............................................................  11187

Query  181    GTACCTCAAATTGTAATCTATTTGTTCCATCGTCTGTCTTGTGGCTAGATTGTAACTTGT  240
Sbjct  11186  ............................................................  11127

Query  241    GACCTCTTTGCCTTGGTCTGTGTCTTTGGGATCGTAATCCCTGGGTAATGTTGTTGTCCT  300
Sbjct  11126  ............................................................  11067

Query  301    CAAGTTGATCACCGCTTGCGACAACGGTTCTACTATCCCCATGTTGTGGACTAACATCAT  360
Sbjct  11066  ............................................................  11007

Query  361    TCTATCTTTGTATTGTAGCGTGTGGCCTATGATTATCCCTTTCTCCGTGTTTGCTATCAT  420
Sbjct  11006  ............................................................  10947

Query  421    TACCTGCCCCGGCTTAATTGTTTGGGGTTTTTCTATCATTATATAATCGTTTCCGGTGCT  480
Sbjct  10946  ............................................................  10887

Query  481    CAGGGGACTAAAAATCACTGGCTCTGTCATGCTTCTAAACACGTGTGTGATTTTCTCTAG  540
Sbjct  10886  ............................................................  10827

Query  541    TGTTATTCTTTGGTGCACTGACTTTACGTGGTTAGCTGTTTTGTCTGACCAATAACCCCC  600
Sbjct  10826  ...........................................................T  10767

Query  601    GCCCA  605
Sbjct  10766  .T...  10762
```

Figure S6. Nucleotide identities between psittacine alphaendornavirus isolate002 sequenced in this study and Helianthus annuus alphaendornavirus (GenBank accession no. NC_040799.1).

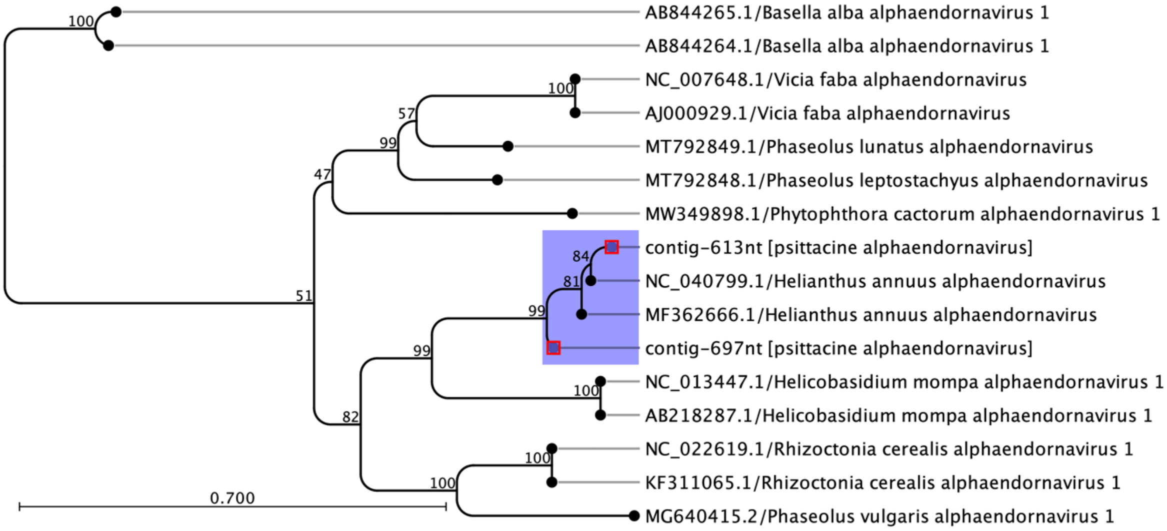

Figure S7. Phylogenetic tree showed the possible evolutionary relationship of psittacine alphaendornavirus detected in this study with other selected alphaendornavirus using partial polyprotein gene. Nucleotides sequences of partial polyprotein gene were aligned with MAFTT (version 7.450) (Katoh and Standley, 2013) in Geneious (version 10.2.2). The ML tree was constructed under the GTR substitution model and 1000 bootstrap re-samplings using tools available in CLC Genomics Workbench (version 9.5.4). The numbers on the left show bootstrap values as percentages, and the labels at branch tips refer to original GenBank accession numbers followed by organism name in parentheses. The subclade related to this study is highlighted as blue background colours.

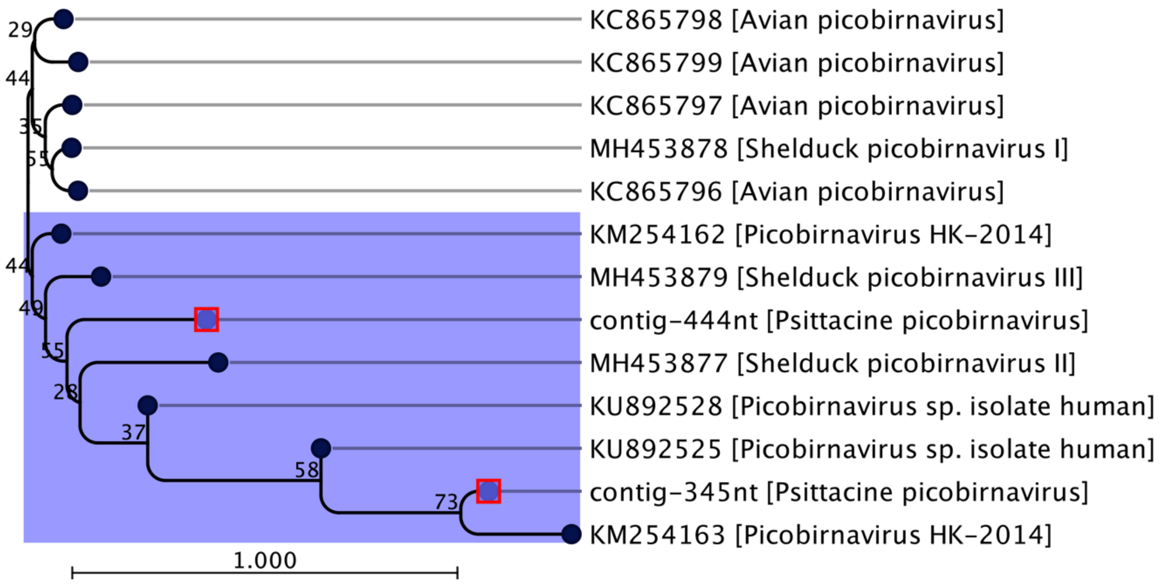

Figure S8. Phylogenetic tree showed the possible evolutionary relationship of psittacine picobirnavirus detected in this study with other selected picobirnavirus partial gene. Nucleotides sequences were aligned with MAFTT (version 7.450) (Katoh and Standley, 2013) in Geneious (version 10.2.2). The ML tree was constructed under the GTR substitution model and 1000 bootstrap re-samplings using tools available in CLC Genomics Workbench (version 9.5.4). The numbers on the left show bootstrap values as percentages, and the labels at branch tips refer to original GenBank accession numbers followed by organism name in parentheses. The subclade related to this study is highlighted as blue background colours.

Table S1. Pairwise identity of representative siadenovirus species against psittacine siadenovirus F strain S10/AU on the basis of complete genome nucleotide sequences

Siadenovirus genomes		1	2	3	4	5	6	7	8	9	10	11
**Psittacine siadenovirus F [MZ364296]**	1											
Psittacine siadenovirus F [MW365934]	2	99.78										
Turkey siadenovirus A [AC_000016]	3	54.65	54.74									
Turkey siadenovirus A [AF074946]	4	54.65	54.74	100								
Penguin siadenovirus A [KP144329]	5	53.73	53.75	63.56	63.56							
Skua siadenovirus A [HM585353]	6	57.04	57.08	60.8	60.8	59.63						
Raptor adenovirus A [EU715130]	7	55.6	55.62	59.5	59.5	58.47	71.23					
Psittacine siadenovirus D [MN687905]	8	54.4	54.4	58.46	58.46	57.35	63.72	62.59				
Psittacine siadenovirus D [MK695679]	9	54.52	54.56	58.4	58.4	57.53	63.59	62.49	89.7			
Psittacine siadenovirus E [MK227353]	10	53.8	54.07	58.56	58.56	57.23	63.83	62.6	75.27	75.24		
Frog siadenovirus A [NC_002501]	11	49.54	49.59	48.47	48.47	48.19	50.49	49.86	48.62	48.68	48.64	

Table S2. Pairwise identity of representative adenovirus against partial penton gene of human adenovirus 2 detected in this study

		1	2	3	4	5	6	7	8	9	10	11
**Human adenovirus 2 [MZ364301/seg:1-958]**	1											
Human adenovirus 2 [MT277585.1/seg:14111-15043]	2	99.7										
Human mastadenovirus C [MH121106.1/esg:14047-14979]	3	99.7	100									
Human mastadenovirus C [MH121105.1/seg:14047-14979]	4	99.7	100	100								
Human mastadenovirus C [MH121104.1/seg:14043-14975]	5	99.7	100	100	100							
Human mastadenovirus C [MH121103.1/seg:14130-15062]	6	99.7	100	100	100	100						
Human adenovirus 2 [EU128937.1/seg:1846-2778]	7	99.7	100	100	100	100	100					
Adenovirus type 2 [J01917.1/seg:14111-15043]	8	99.7	100	100	100	100	100	100				
Human adenovirus 2 [MN513342.1/seg:14107-15039]	9	99.6	99.9	99.9	99.9	99.9	99.9	99.9	99.9			
Human mastadenovirus C [MH121109.1/seg:14039-14971]	10	99.6	99.9	99.9	99.9	99.9	99.9	99.9	99.9	100		
Human mastadenovirus C [MH121102.1/seg:14047-14979]	11	99.6	99.9	99.9	99.9	99.9	99.9	99.9	99.9	100	100	

Table S3. Pairwise identity of representative adenoviruses against partial capsid protein precursor pIIIa gene of human mastadenovirus C detected in this study

		1	2	3	4	5	6	7	8	9	10	11
**Human mastadenovirus C [MZ364303/seg:1-435]**	1											
Mutant Human adenovirus 2 [MT277585.1/seg:13537-13971]	2	98.9										
Human adenovirus 2 [MN513342.1/seg:13533-13967]	3	98.9	100									
Human mastadenovirus C [MH121111.1/seg:13533-13967]	4	98.9	100	100								
Human mastadenovirus C [MH121109.1/seg:13468-13902]	5	98.9	100	100	100							
Human mastadenovirus C [MH121106.1/seg:13475-13909]	6	98.9	100	100	100	100						
Human mastadenovirus C [MH121085.1/seg:13453-13887]	7	98.9	100	100	100	100	100					
Human adenovirus 2 [J01917.1/seg:13537-13971]	8	98.9	100	100	100	100	100	100				
Human mastadenovirus C [MH121095.1/seg:13465-13899]	9	98.2	99.3	99.3	99.3	99.3	99.3	99.3	99.3			
Human mastadenovirus C [MH121089.1/seg:13538-13972]	10	98.2	99.3	99.3	99.3	99.3	99.3	99.3	99.3	100		
Human adenovirus 1 [MN737436.1/seg:13561-13995]	11	98.2	99.3	99.3	99.3	99.3	99.3	99.3	99.3	100	100	

Table S4. Pairwise identity of representative adenoviruses against partial encapsidation protein 52K gene of human mastadenovirus C detected in this study

		1	2	3	4	5	6	7	8	9	10	11
**Human mastadenovirus C [MZ364302/seg:1-430]**	1											
Human mastadenovirus C [MK836309.1/seg:11519-11908]	2	99										
Human adenovirus 2 [MT277585.1/seg:11507-11896]	3	99	100									
Human mastadenovirus C [MH121111.1/seg:11503-11892]	4	99	100	100								
Human mastadenovirus C [MH121106.1/seg:11445-11834]	5	99	100	100	100							
Human mastadenovirus C [MH121105.1/seg:11445-11834]	6	99	100	100	100	100						
Human mastadenovirus C[MH121090.1/seg:11458-11847]	7	99	100	100	100	100	100					
Human adenovirus 6 [LC068720.1/seg:11475-11864]	8	99	100	100	100	100	100	100				
Human adenovirus 6 [LC068712.1/seg:11514-11903]	9	99	100	100	100	100	100	100	100			
Human adenovirus C [KF951595.1/seg:11511-11900]	10	99	100	100	100	100	100	100	100	100		
Human adenovirus 1 [MN737436.1/seg:11531-11920]	11	99	100	100	100	100	100	100	100	100	100	
